# Elucidating the Diagnostic Complexity of Round Cell Sarcoma with EWSR1-CREM Fusion: A Comprehensive Case Study

**DOI:** 10.3390/medicina60040544

**Published:** 2024-03-27

**Authors:** Hao Yen, Jian-Liang Chou, Yao-Feng Li, Der-Shiun Wang

**Affiliations:** 1 Department of Pathology, Tri-Service General Hospital, National Defense Medical Center, Taipei 114, Taiwan; kastyplanet@gmail.com; 2 Instrument Center, Department of Research and Development, National Defense Medical Center, Taipei 114, Taiwan; arvin0937@gmail.com; 3 Department of Pediatrics, Tri-Service General Hospital, National Defense Medical Center, Taipei 114, Taiwan

**Keywords:** EWSR1, sarcoma, EWSR1-CREM fusion, EWSR1-CREB family, ALK protein, ALK, TSO500

## Abstract

Sarcomas, particularly undifferentiated small round cell sarcomas of bone and soft tissue, pose significant diagnostic challenges due to their nonspecific morphology and the necessity for comprehensive molecular analyses. This paper discusses a rare case of round cell sarcoma exhibiting the EWSR1-CREM fusion, offering insights into the complexities of its diagnosis and management. The patient, a 15-year-old female with a history of Type 1 diabetes, presented with persistent right thigh tenderness and swelling. MRI revealed a large necrotic mass in the retroperitoneal region. Histological analysis showed a well-demarcated tumor with diverse cellular morphologies and distinct necrotic areas. Immunohistochemical (IHC) tests identified dot-like staining for Desmin and Vimentin but negative results for several markers, including Cytokeratin and CD45. Strong ALK positivity was noted. Next-generation sequencing with the Illumina TruSight™ Oncology 500 assay revealed the fusion gene EWSR1-CREM, along with benign and uncertain mutations in other genes. The tumor’s morphology and immunoprofile, along with molecular findings, led to a diagnosis of round cell sarcoma with EWSR1-CREM fusion. This case adds to the spectrum of tumors associated with this fusion, often presenting diverse morphologies. The rarity of EWSR1-CREM fusion sarcomas poses a challenge in treatment, highlighted by the development of pulmonary metastases and disease progression after surgical excision in this patient despite the lack of an effective targeted therapy. In conclusion, this case emphasizes the need for a multidisciplinary diagnostic approach in complex sarcomas and highlights the importance of continued research on rare sarcomas, their genetic underpinnings, and potential therapeutic targets.

## 1. Introduction

Sarcoma, a diverse and complex group of malignancies, presents a significant challenge in the realm of pathological diagnosis and treatment. Among these, undifferentiated small round cell sarcomas of bone and soft tissue are very challenging, due to the lack of a unique morphology and the requirement of intensive molecular tools; characterized by small round blue cells, these sarcomas are particularly notorious for their diagnostic ambiguities and aggressive clinical course. This paper aims to summarize a potentially unique entity of sarcoma exhibiting the rare EWSR1-CREM fusion, thereby contributing to the growing body of knowledge in sarcoma pathology. Through a detailed case presentation, we highlight the critical role of comprehensive histological and immunohistochemical analysis, supplemented by advanced molecular techniques, in reaching an accurate diagnosis. Additionally, this manuscript endeavors to find the importance of integrating clinical, histopathological, and molecular data in the management of such complex sarcomas. Our objective is to share valuable insights and experiences from this case to aid pathologists, oncologists, and researchers in better understanding and discussing the possible effective management of similar challenging cases of sarcoma.

## 2. Case Report

**Figure 1:**
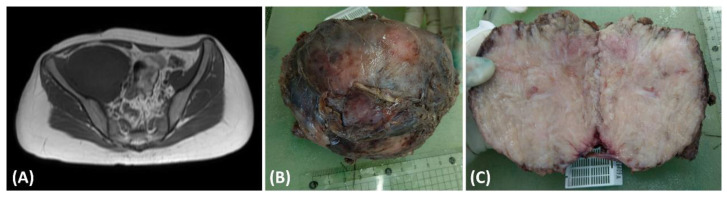
(**A**) The T1-weighted magnetic resonance imaging (MRI) scan exhibited a substantial intraabdominal mass originating from the retroperitoneal space, demonstrating invasion into adjacent tissues. (**B**) The dimensions of the excised tumor were 13.5 × 9.2 cm, characterized by fibrotic encapsulation and a firm texture. (**C**) Examination of the cut surface of the resected tumor disclosed a composition predominantly fleshy and fibrotic, interspersed with areas of focal central necrosis.

This case pertains to a 15-year-old female patient with a preexisting medical history of Type 1 diabetes mellitus characterized by suboptimal glycemic control. The patient complained of tenderness and swelling in the right thigh, persisting for 5 months, significantly impacting her nocturnal rest. Seeking medical assistance, she sought evaluation in the pediatric outpatient department, where laboratory investigations revealed elevated C-reactive protein (CRP) levels (5.95 mg/dL) and ESR levels (119 mm/h), and the presence of proteinuria (2+ in urinalysis). In light of these clinical findings, a pediatric nephrologist arranged abdominal sonography for initial examination to rule out kidney lesions and incidentally found one soft tissue mass behind the urinary bladder. The nephrologist then conducted a magnetic resonance imaging (MRI) examination (Figure 1A), revealing a substantial necrotic mass measuring 13.5 × 9.2 cm in the retroperitoneal region (Figure 1B) involving the right psoas and iliac muscle. Upon suspicion of malignancy, a non-contrast chest computed tomography was conducted to surveil potential tumor origins. The scan revealed multiple soft tissue nodules, with the largest measuring up to 3.84 cm × 2.71 cm. These imaging findings strongly suggested the presence of multiple pulmonary metastases. Subsequently, the patient underwent surgical excision of the retroperitoneal tumor, which was removed for disease control and pathologic examinations. The cut surface of the tumor presented a fleshy appearance with a mildly firm consistency (Figure 1C).

**Figure 2:**
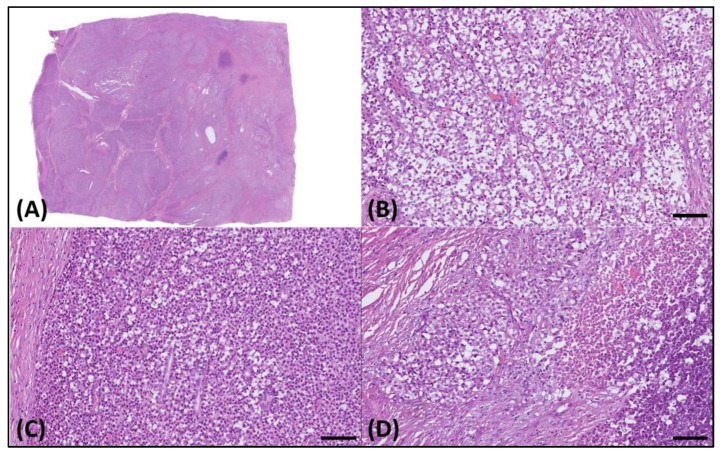
Histology of the resected tumor. (**A**) Microscopically, this was a well-demarcated tumor. (**B**) The tumor cells exhibited varied morphologies, including round cell, epithelioid, and (**C**) plasmacytoid features, with clear cytoplasm and (**D**) notable necrotic areas. The scale bar is 100 µm.

Microscopically, the sample presented as a relatively well-demarcated tumor with focal infiltrative features along its margins. It predominantly exhibited a sheet-like growth pattern, delineated by dense fibrotic strands (Figure 2A), interspersed with regions displaying an alveolar pattern. The tumor comprised cells exhibiting diverse morphological characteristics, such as round cell and epithelioid structures with clear cytoplasm (Figure 2B), along with sporadic plasmacytoid traits (Figure 2C). Furthermore, distinct areas of tumor necrosis were also discernible (Figure 2D).

**Figure 3:**
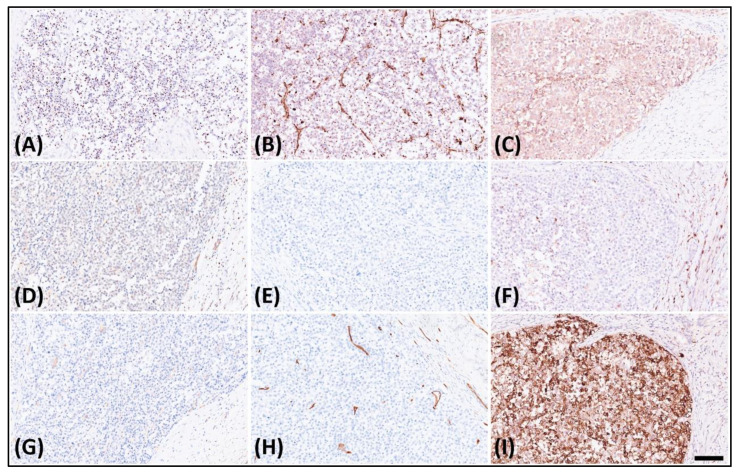
Immunohistochemistry of the tumor revealed. (**A**) Dot-like staining of Desmin, (**B**) dot-like staining of Vimentin, (**C**) faint cytoplasmic staining of Synaptophysin, (**D**) faint nuclear staining of WT1’s C-terminal, (**E**) negative staining of Cytokeratin, (**F**) negative staining of CD45, (**G**) negative staining of CD99, (**H**) negative staining of CD34, and (**I**) strongly diffuse staining of ALK.

**Figure 4:**
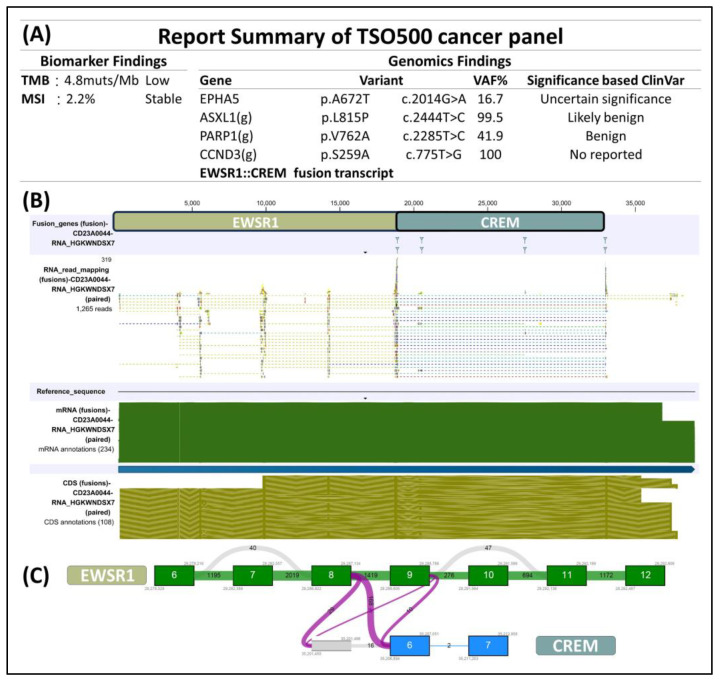
(**A**) Next-generation sequencing by using Illumina TSO500 identified EWSR1-CREM fusion in the tumor. Additional benign and uncertain mutations in ASXL1, PARP1, EPHAS, CCND3 were found. (**B**) Upon closer examination of the EWSR1-CREM fusion, we were able to determine (**C**) that this tumor originated from a fusion event involving exon 8 of the gene EWSR1 and exon 6 of the gene CREM.

The immunohistochemical analysis displayed dot-like staining for Desmin (Figure 3A) and Vimentin (Figure 3B), with faint cytoplasmic Synaptophysin (Figure 3C) and nuclear WT1’s C-terminal staining (Figure 3D). The tumor tested negative for Cytokeratin (Figure 3E), CK18, EMA, claudin-4, myogenin, myoD1, CD45 (Figure 3F), CD99 (Figure 3G), CD34 (Figure 3H), CD56, PAX8, GATA-3, and MUC4 but retained MTAP, BAP1, INI-1, and BRG-1. It was also negative for Melan A, HMB45, SOX10, NKX2-2, ERG, FLI-1, INSM-1, ETV4, BCOR, and SATB2. Notably, in the search for targetable biomarkers, strong ALK positivity was detected in the tumor (Figure 3I). Morphology and immunoprofile suggested a diagnosis of desmoplastic small round cell tumor. To confirm the diagnosis, we utilized the Illumina TruSight™ Oncology 500 (TSO500) assay for next-generation sequencing. In the obtained data, the RNA sequencing FASTQ file was analyzed by using the TSO500 RNA Analysis workflow integrated into the Qiagen CLC Genomics Workbench (v23). In summary, the FASTQ file underwent processing steps including read trimming, UMI read creation, and quality checking. Subsequently, it was aligned with the RelSeq GRCh38 database to detect and refine fusion genes. The results are presented in Tracks (fusion) and a fusion report. The panel revealed mutations in several genes, including ASXL1, PARP1, EPHAS, and CCND3. Consultation with the ClinVar database (https://www.ncbi.nlm.nih.gov/clinvar/ (accessed on 28 January 2024)) indicated that the mutations in ASXL1 and PARP1 were benign, whereas the significance of mutations in EPHAS and CCND3 remained uncertain or unreported (Figure 4A). This targeted, hybrid-capture method identified the fusion gene EWSR1-CREM (Figure 4B), with the fusion event involving exon 8 of the gene EWSR1 and exon 6 of the gene CREM. Given the tumor’s invasive characteristics, with pulmonary metastases having been found in the subsequent image studies, the conclusive diagnosis was a round cell sarcoma characterized by the EWSR1-CREM fusion.

After establishing the diagnosis in this case, the pediatric oncologist proposed administering systemic chemotherapy to the patient. However, the patient and her legal guardian declined this recommendation due to concerns about potential adverse effects of the treatment. In addition to managing glycemic levels with insulin, the patient opted for weekly cell therapy for six cycles and underwent concurrent local radiotherapy targeting the pulmonary metastatic site for four cycles, with doses of 4, 6, 8, and 10 Gy, respectively, in order to trigger a possible synergy effect of cell therapy. Regrettably, the patient’s condition progressed, manifesting local recurrence in the right retroperitoneal region and bone metastasis, as revealed in subsequent MRI scans conducted 6 months later. She is currently being treated as an outpatient, receiving palliative radiotherapy delivering 50 Gy in 25 fractions to the recurrent tumor site for pain control, along with oral Oxycontin and a Fentanyl transdermal patch.

## 3. Discussion

### 3.1. Histological Differential Diagnosis and Education Points

This case describes a clear and round cell soft tissue neoplasm of the retroperitoneum; however, despite comprehensive histological and immunohistochemical studies, it remains difficult to definitively diagnose and classify. The excised specimen showed a well-circumscribed tumor with a focal permeated border in the retroperitoneum and additional morphologies of hyalinized fibrotic septa surrounding clear, focal epithelioid, and plasmacytoid tumor cell nests, as well as central necrosis. The morphology of the tumor is similar to soft tissue sarcomas of uncertain type, such as Ewing sarcoma and clear cell sarcoma. However, the immunohistochemical stains did not support the above diagnoses except for the specific pattern of dot-like staining of Desmin and Vimentin. These findings support the diagnosis of desmoplastic small round cell tumor [1].

According to the World Health Organization (WHO) classification, a desmoplastic small round cell tumor is a malignant mesenchymal neoplasm with small round tumor cells associated with prominent stromal desmoplasia and polyphenotypic differentiation, as well as the genetic fusion of EWSR1-WT1 [2]. Despite the specific finding in the tumor cells of Desmin and Vimentin, the aberrant features of tumor cells having epithelioid morphology, focal eosinophilic cytoplasm, and negative staining of Cytokeratins point at the novel entity of a rare tumor in our case. Thus, panels of immunohistochemical studies were performed for ruling out relevant cases considered, as demonstrated above (Table 1). Also, strong positive staining of ALK and faint nuclear staining of WT1 antibody of the C terminus do not represent strong supportive evidence for the precise diagnosis of a desmoplastic small round cell tumor. Therefore, we performed Next-generation sequencing by using Illumina TruSight™ Oncology 500, and the fusion gene EWSR1-CREM was detected in the specimen. The final diagnosis was a round cell sarcoma with EWSR1-CREM fusion.

### 3.2. Summary of the Current Literature

The gene EWSR1 is a member of the TET family, a group of genes identified in several translocation-associated sarcomas [3]. The CREB family transcription factors include ATF1, CREB1, and CREM, constituting a group of the basic leucine zipper (bZIP) superfamily and encoding several events in human tumors [4,5]. The two families’ fusion tumors are frequently discovered in several entities, such as angiomatoid fibrous histiocytoma, clear cell sarcoma in the gastrointestinal tract, primary pulmonary myxoid sarcoma, and hyalinizing clear cell carcinoma of the salivary gland [5].

Previous studies, including those by Kao et al. [5], highlight that tumors associated with the EWSR1-CREM fusion typically exhibit a lobulated architecture, comprising uniform ovoid-to-round cells organized in cord-like or reticular patterns against a myxoid background. These tumors are commonly found in children and young adults, with a frequent occurrence in the intracranial region. Conversely, a separate analysis of 13 cases identified EWSR1-CREM fusion neoplasms in mesothelial-lined cavities, characterized by distinctive epithelioid, round, and rhabdoid morphologies, affecting a wide age range of patients [6]. Yoshida et al. reported the EWSR1-CREM fusion in diverse tumor types, including a clear cell sarcoma of soft tissue, three angiomatoid fibrous histiocytomas in pulmonary and extremity locations, and two undefined tumors in the abdominal cavity and chest wall (with one presenting a spindle morphology and the other a round cell morphology) [4]. Komatsu et al. [7] documented a pulmonary mesenchymal tumor with clear cytoplasm and an alveolar, nested structure. Additionally, Cui et al. [8] described a gastric tumor characterized by mesenchymal components with ovoid nuclei and scant cytoplasm. Other literature reviews have noted various morphological features in classifiable tumors, such as polygonal cells with indistinct borders [9] and clear or eosinophilic epithelioid cells within fibrous or hyalinized stroma separating the tumor nests [10,11,12,13,14] (Table 2). These findings collectively demonstrate a wide spectrum of tumor morphologies linked to the EWSR1-CREM fusion, suggesting that this fusion type might be present in different currently classified tumor entities.

**Table 1:** Summary of morphologies, IHC studies, and molecular findings of the indexed case and relevant diagnoses [1,14,15].

	Indexed Case	Desmoplastic Small Round Cell Tumor [1]	Clear Cell Sarcoma [15]	Ewing Sarcoma
Hematoxylin and eosin morphology	Clear, epithelioid, rhabdoid cytoplasm, hyperchromatic nuclei; hyalinized, fibrotic stroma	Small uniform, hyperchromatic nuclei, scant cytoplasm; prominent desmoplastic stroma	Nested, fascicular architecture with epithelioid-to-plump spindle cells, eosinophilic cytoplasm, vesicular nuclei; thin fibrous stroma	Small round nuclei with stippled chromatin, indistinct cytoplasmic membrane, and neuroectodermal differentiation
CK(AE1/AE3)	−	+	−	−/+
CD99	−	+	N/A	+
Desmin	Dot-like staining	Dot-like staining	−	N/A
Vimentin	Dot-like staining	Dot-like staining [14]	+	+
WT1’s C terminus	Faint nuclear staining	Strong nuclear staining	−	−
NKX2-2	−	−	−	+
S100	−	−	+	+/−
HMB45	−	−	+	−
Synaptophysin	+	+/−	−/+	+/−
ALK protein	+	−	N/A	N/A
Molecular findings	EWSR1-CREM fusion	EWSR1-WT1 fusion	EWSR1-ATF1/CREB1 fusion	FET-ETS fusion

+, positive; −, negative; N/A, not applicable; +/− majority of cases may be positive; −/+, minority of cases may be positive.

### 3.3. Therapeutics

Currently, effective treatments for EWSR1-CREM fusion sarcoma are limited, primarily due to its rarity on a global scale. In our pursuit of identifying targetable biomarkers, we conducted various immunohistochemical tests, revealing a strong, diffuse staining pattern for ALK protein. Notably, this finding does not necessarily indicate the presence of an ALK fusion [6]. According to studies by Agaimy et al. [14] and Cheah et al. [16], ALK protein expression is believed to be a downstream target of EWSR1 fusion proteins, leading to positive ALK immunohistochemical stains. However, this hypothesis remains speculative and requires further similar case studies. In a relevant study, Subbiah et al. [17] reported on a 27-year-old female diagnosed with gastrointestinal neuroectodermal tumor (GNET) harboring the EWSR1-CREB1 fusion with liver metastasis. The patient was treated with the c-Met/ALK inhibitor Crizotinib and the multi-kinase VEGF inhibitor Pazopanib, achieving near-complete response in 1.5 years. This study suggests potential treatment options for tumors harboring the EWSR1-CREB fusion family in the future. Nonetheless, the efficacy of targeted therapies in treating this condition remains uncertain and requires further investigation due to limited case studies.

## 4. Conclusions

This case underscores the complexity and challenges inherent in diagnosing and managing round cell sarcomas, particularly those with rare genetic alterations like the EWSR1-CREM fusion. The meticulous integration of histological, immunohistochemical, and molecular diagnostic techniques was pivotal in arriving at a definitive diagnosis in this case. Our findings reiterate the importance of considering a broad differential diagnosis when encountering round cell sarcomas, especially in atypical presentations. Furthermore, the discovery of the EWSR1-CREM fusion in this tumor expands our understanding of the genetic landscape of round cell sarcomas, suggesting that this fusion might be more prevalent across different sarcoma entities than previously recognized. This case also highlights the need for continued research into targeted therapies for rare sarcomas, as evidenced by the discovery of ALK protein expression in this tumor. The aggressive nature associated with these tumors, as demonstrated by the pulmonary metastases in our patient when the tumor was identified at the outset, alongside the limited treatment effect of cell therapy combined with radiotherapy and subsequent local recurrence within 6 months after complete surgical excision, call for a more robust, multimodal approach in their management. The c-MET/ALK inhibitor and multi-kinase VEGF inhibitor may represent a potential treatment option for tumors harboring the EWSR1-CREB fusion family, yet further investigation is required. In conclusion, our case contributes valuable insights into the complex pathology of round cell sarcomas, advocating for a comprehensive diagnostic approach and underscoring the necessity for ongoing research in this field.

## Figures and Tables

**Table 2:** Clinical pathological summary of tumors harboring the EWSR1-CREM fusion.

Reference	Age/Sex	Site	Morphology	Positive IHC
Indexed case	15/F	Retroperitoneum	Clear, epithelioid, rhabdoid cytoplasm, separated by hyalinized, fibrotic stroma	Demin, Vimentin (dot-like staining), Synaptophysin, and ALK
Dickson et al. (2018) [9]	48/M	Dorsal tongue	Small nodules of polygonal cells, indistinct cell borders, and prominent slit-like spaces (ECT)	Non
Chapman et al. (2018) [12]	68/F	Tongue	Infiltrating tumor in hyalinized stroma with associated lymphocytic reaction (HCCC)	CK5 and p63
	75/F	Lung	Hyalinized stroma with focal clear cells (HCCC)	CK7, CK14, and p63
	62/M	Nasopharynx	Cords and nests of tumor cells in hyalinized stroma (HCCC)	CK7, CK14, and p63
Yoshida et al. (2019) [4]	48/F	Left hand	Monomorphic nuclei with prominent nucleoli and amphophilic-to-clear cytoplasm (CCS)	S100 protein, HMB45, Melan A, and SOX10
	47/M	Lung	Myxoid variant, showing multinodular, predominantly myxoid growth populated by uniform round-to-spindle cells in a reticular pattern (myxoid AFH)	EMA, Desmin (2 of the cases were positive for ALK)
	50/M	Finger		
	54/M	Hand		
	15/M	Abdominal cavity	Hypercellular storiform-to-whorled pattern, small oval-to-spindle cells with fine chromatin, and delicate intercellular collagen deposition	Vimentin, AE1/AE3, EMA (focal), CD56, CD34 (focal), ALK (focal), DOG1 (focal), and Synaptophysin (focal)
	63/F	Left chest wall	Small round cells with fine chromatin, scant eosinophilic cytoplasm, fibrillary stroma, vague trabecular-to-reticular pattern, and perivascular pseudorosettes	CD99, Synaptophysin, Vimentin, CD56, MUC4, and EMA (focal)
Argani et al. (2020) [6]	9/M	Adrenal	Epithelioid, tubular structures; cystic, lymphoid cuff; calcifications	AE1/3, Cam5.2, EMA (focal), WT1(focal), inhibin, Desmin (focal), and CD99 (weak)
	47/F	Mesocolon	Epithelioid and spindle cells, cystic, lymphoid cuffs	CK (focal)
	25/M	Gastric fundus	Epithelioid and round cells, cystic, lymphoid cuffs	EMA (focal) and WT1 (rare)
	44/F	Pleura	Epithelioid and round, rhabdoid cells, cystic, pseudopapillary	AE1/3 (focal); EMA and inhibin (focal)
	20/F	Peri-rectal	Epithelioid and spindle cells, myxoid	CK (focal) and EMA (focal)
	14/F	Thigh	Epithelioid and rhabdoid cells, cystic	CK (focal), EMA (focal), and CD99
	29/M	Kidney	Round and epithelioid, cystic	CK (focal)
Komatsu et al. (2020) [7]	56/M	Lung	Clear cell, alveolar and nested pattern	Vimentin, Synaptophysin, and ALK(5A4)
Agaimy et al. (2022) [14]	55/M	Kidney	Epithelioid cells, few rhabdoid cells, communicating nests, trabeculae, and fibrous stroma	Vimentin, EMA, AE1/AE3, Cytoplasmic Alk, and variable MUC4
Cui et al. (2022) [8]	69/F	Stomach	Bland oval cells with scanty cytoplasm and monomorphic nuclei	CD10, CD117, and CD56 (patchy)
Philippa Li et al. (2023) [13]	41/F	Right great toe	Large nuclei, prominent nucleoli, and abundant cytoplasm with focal clear cell change (CCS)	S100, HMB45, SOX10, and Melan-A
Sijian Li et al. (2023) [11]	7/F	Left ovary	Epithelioid cytoplasm, round or oval nuclei, and fibrous/fibromyxoid stroma (OEMPNST)	SOX10, S-100, INI-1 (retained), α-inhibin, and H3k27me3 (retained)
Javaid et al. (2023) [10]	36/M	Neck	Polygonal cells, pale eosinophilic cytoplasm, and solid growth (paraganglioma)	Synaptophysin, chromogranin, and S100 (sustentacular cells)

ECT: ectomesenchymal chondromyxoid tumor; HCCC: hyalinizing clear cell carcinoma; CCS: clear cell sarcoma; OEMPNST: ovarian epithelioid malignant peripheral nerve sheath tumor.

## Data Availability

Data are contained within the article.
